# Prevalence of infection by *Bartonella* spp. in patients with psoriasis^☆^^☆☆^

**DOI:** 10.1016/j.abd.2020.07.004

**Published:** 2020-11-20

**Authors:** Luciene Silva dos Santos, Marina Rovani Drummond, Renata Ferreira Magalhães, Marilene Neves da Silva, Patricia Andreia Rodrigues Ferreira, Paulo Eduardo Neves Ferreira Velho

**Affiliations:** a Laboratory of Applied Research in Dermatology and Bartonella Infection, Faculty of Medicine, Universidade Estadual de Campinas, Campinas, SP, Brazil; b Dermatology Division, Department of Clinical Medicine, Faculty of Medical Sciences, Universidade Estadual de Campinas, Campinas, SP, Brazil

*Dear Editor,*

Psoriasis (Ps) is a chronic multisystem inflammatory disease that, in addition to the genetic factor, has other triggers such as emotional stress, nutritional deficit, endocrine problems, and infections. The activation of immune system cells is considered an important factor in the pathogenesis of Ps, and several infectious agents have been related to this activation. To modulate the immune response in patients with Ps, the systemic treatment of the disease may be based on immunosuppressive drugs, which facilitates the spread of opportunistic infections.^1^

Bacteria of the genus *Bartonella* are fastidious Gram-negative cocobacilli distributed worldwide (Fig. 1). Currently, the genus has 45 species and subspecies, of which at least 17 are capable of infecting humans. Most of these bacteria are transmitted by hematophagous arthropods, and some of their reservoirs are domestic animals, mainly dogs and cats. Although they have been neglected, the number of studies on *Bartonella* spp. is increasing, as well as the recognition of their importance. These agents have been linked to a wide spectrum of clinical manifestations, ranging from asymptomatic infection to life-threatening conditions, such as endocarditis.^2^

**Figure 1:**
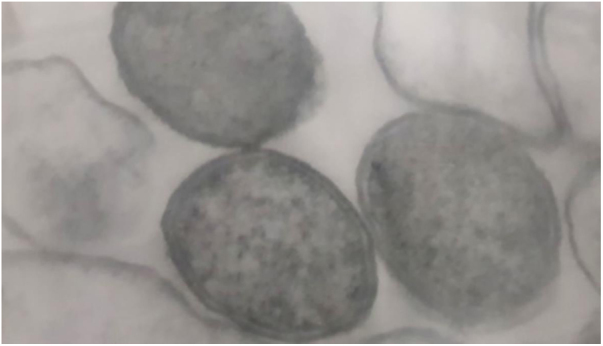
Transmission electronmicroscopy photomicrograph of a colony of *Bartonella henselae* (ATCC 49793) after 45 days of growth on blood-enriched agar: cocoid and electrondense bacteria with a tri-laminar wall, 50,000×.

There are no diagnostic tests with sufficiently high sensitivity and specificity. In addition, bartonellosis is not included in the diagnostic hypotheses by most physicians, which contributes to the underdiagnosis of these infections.^2^ This study aimed to assess the prevalence of *Bartonella* spp. infection through molecular and microbiological tests in Ps patients and a control group of volunteers.

The project was approved by the Institutional Research Council of the Universidade Estadual de Campinas (University of Campinas), under protocol CAAE: 48057415.5.0000.5404.

Blood samples were obtained from 30 Ps patients over 18 years of age, with mild to severe manifestations in different therapeutic regimens who agreed to participate in the study, as well as 30 volunteers – Unicamp students or employees over 18 years of age who denied clinical symptoms, were not pregnant, and agreed to participate in the study.

The samples were processed as summarized in Fig. 2. Liquid enrichment cultures and solid cultures were performed as previously described.^3^ From whole blood and culture samples, DNA was extracted using the QIAmp DNA Mini Kit (Qiagen®).

**Figure 2:**
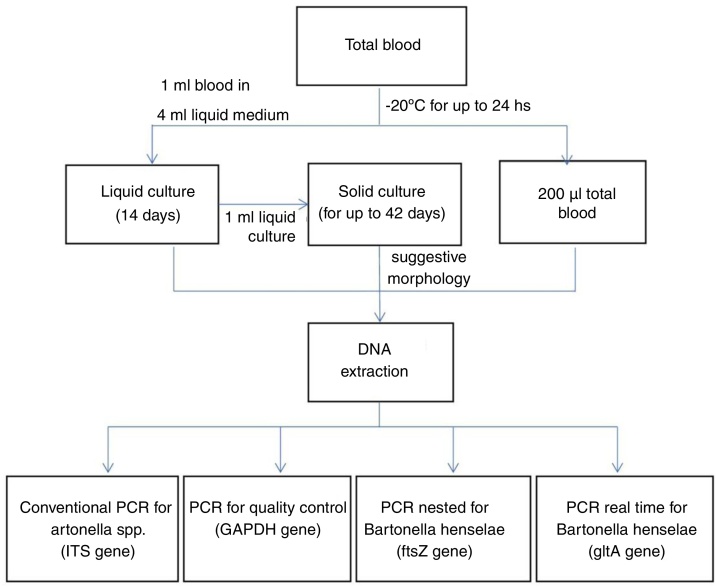
Flowchart of the procedures performed.

From the obtained DNA, genus-specific conventional PCRs (*ITS* region) and *Bartonella henselae*-specific PCRs were performed: double amplified PCR (nested) for the *ftsZ* region and real-time PCR for the *gltA* region. The quality of the extracted DNA and the absence of amplification inhibitors were tested using conventional PCR for the *GAPDH* gene.

*B. henselae* DNA was detected in 20% (6/30) of Ps patients and in 10% (3/30) of healthy volunteers who denied symptoms at the time of blood sample collection (Table 1). Using Fisher's exact test, no statistical difference was observed between the two groups (*p* = 0.23).

**Table 1:** Results of positive samples.

Positives	Conventional PCR liquid culture	Nested PCR whole blood	Nested PCR liquid culture	PCR whole blood	Real-time PCR liquid culture	Real-time PCR solid culture
PS5	–	–	–	+	–	–
PS16	–	–	**+**	–	–	–
PS18	–	–	–	**+**	–	–
PS20	–	–	**+**	**+**	**+**	–
PS28	–	–	–	–	–	**+**
PS30	–	–	**+**	–	–	–
CG13	**+**	**+**	–	–	–	–
CG36	–	**+**	–	–	–	–
CG40	−	**+**	–	–	–	–

PS, patients; CG, control group.

Ps is a multifactorial, inflammatory, and immune-mediated disease. Although there is no consensus on the exact mechanisms of action in its pathogenesis, there is strong evidence that external factors, such as super antigens, have a great capacity to stimulate the inflammatory response of the disease.^1^ Microorganisms have been associated with Ps (including β-hemolytic streptococci, *Staphylococcus aureus*, *Porphyromonas gingivalis*, *Candida albicans*, *Chlamydia psittac*i, human immunodeficiency virus, and hepatitis C virus), but there is limited evidence that antimicrobial therapy has any direct benefit in crisis prevention. Ps is independently associated with a higher risk of serious infections, which is increased by the use of immunomodulatory treatments.^1^

Infection by *Bartonella* spp. was documented in 3.2% of 500 blood donors using a single conventional genus-specific PCR, from samples of liquid and solid culture.^4^

*Bartonella* spp. was detected in patients with Ps and psoriatic arthritis (PsA). One patient with Ps presented with cat-scratch disease during treatment with adalimumab, and another patient with PsA presented mesenteric lymphadenopathy and splenic abscesses. Symptomatic infection by *Bartonella* spp. was detected in other patients who were receiving treatment with immunobiologicals.^5^

One in five patients with Ps and one in ten healthy volunteers presented infection by *B. henselae*. Despite the lack of statistical difference when compared with the control group, this information is important when considering the high prevalence of infection in patients with Ps and even in the control group. Attention is needed for any patient who requires immunobiological treatment or other immunosuppressive drugs and who presents with possible expressions of infection by *Bartonella* spp., such as fever of undetermined origin, cryptogenic hepatitis, lymph node enlargement, endocarditis, sepsis, and graunlomatous or angioproliferative reactions. Further studies are needed to assess whether infection by *Bartonella* spp. may worsen Ps expression and the risks of this infection associated with immunosuppressive treatments.

## Financial support

CNPq doctoral scholarship 170501/2018-3 (Santos, LS); Fapesp Postdoctoral scholarship 2018/12565-6 (Drummond, MR); CNPq productivity grant 301900/2015-9 (Velho, PENF) and Fundo de Apoio à Dermatologia (Funaderm)/Sociedade Brasileira de Dermatologia.

## Authors’ contributions

Luciene Silva dos Santos: Approval of the final version of the manuscript; design and planning of the study; drafting and editing of the manuscript; collection, analysis, and interpretation of data.

Marina Rovani Drummond: Approval of the final version of the manuscript; design and planning of the study; drafting and editing of the manuscript; collection, analysis, and interpretation of data; effective participation in research orientation; critical review of the literature; critical review of the manuscript.

Renata Ferreira Magalhães: Approval of the final version of the manuscript; collection, analysis, and interpretation of data.

Marilene Neves da Silva: Collection, analysis, and interpretation of data.

Patricia Andreia Rodrigues Ferreira: Design and planning of the study.

Paulo Eduardo Neves Ferreira Velho: Statistical analysis; approval of the final version of the manuscript; design and planning of the study; elaboration and writing of the manuscript; obtaining, analyzing, and interpreting the data; effective participation in research orientation; intellectual participation in propaedeutic and/or therapeutic conduct of studied cases; critical review of the literature; critical review of the manuscript.

## Conflicts of interest

None declared.
